# Peptidoglycan-Like Components in Z-100, Extracted from *Mycobacterium tuberculosis* Strain Aoyama B, Increase IL-12p40 via NOD2

**DOI:** 10.1155/2022/3530937

**Published:** 2022-06-22

**Authors:** Takayuki Horii, Yuki Orikawa, Yuta Ohira, Runa Eta, Uiko Tominaga, Takanori Sato, Takao Tanaka

**Affiliations:** Central Research Laboratories, Zeria Pharmaceutical Co., Ltd., 2512-1, Numagami, Oshikiri, Kumagaya City, 360-0111 Saitama, Japan

## Abstract

**Background:**

Z-100 is a hot-water extract of the human-type *Mycobacterium tuberculosis* strain Aoyama B. While Z-100's macrophage-mediated immunomodulatory effects have been reported, the mechanistic details have not been fully clarified. Here, we studied the immunomodulatory effects of Z-100 on mouse bone marrow-derived cells, human CD14^+^ cells, and skin.

**Methods:**

Mouse bone marrow-derived cells and CD14^+^ cells were cultured in the presence of granulocyte-macrophage colony-stimulating factor, differentiated into macrophage-like cells, and then stimulated with Z-100. Furthermore, since Z-100 is subcutaneously administered clinically, we injected Z-100 into mice and measured gene expression in the skin.

**Results:**

While Z-100 stimulation increased the production of interleukin- (IL-) 12p40 and IL-1*β* in mouse bone marrow-derived macrophages, levels of IL-1*β* were low. In contrast, TNF-*α* production did not increase. Meanwhile, stimulation of human CD14^+^ cells with Z-100 increased production of IL-12p40, TNF-*α*, and IL-1*β*. Because Z-100 appeared to have the most stable effect on IL-12p40, we examined the components of Z-100 that induce IL-12p40 production. We found that Z-100 contained peptidoglycan-like components. In addition, an siRNA study showed that Z-100 increased the production of IL-12p40 via nucleotide-binding oligomerization domain 2 (NOD2). Further, subcutaneous administration of Z-100 to mice significantly elevated expression of IL-12p40 and IL-1*β* and showed a trend towards increasing TNF-*α* in the skin.

**Conclusion:**

Z-100 induced the production of immunomodulatory cytokines from various types of macrophages and specifically increased IL-12p40 production through peptidoglycan-like components via NOD2.

## 1. Introduction

Z-100 is a hot-water extract of human-type *Mycobacterium tuberculosis* strain Aoyama B that is clinically marketed for the treatment of leukopenia in Japan (Ancer®) and is under development as a treatment for cancer. Z-100 is thought to be an immunomodulator, with several immune-mediated effects having been reported in previous studies. Oka et al. showed that the metastasis-suppressing effect of Z-100 was abolished in IL-12p40 knockout (KO) mice and that Z-100 induced the production of interferon- (IFN-) *γ* and interleukin- (IL-) 2 from the spleen and IL-12 from peritoneal macrophages of *Bacille Calmette-Guerin-* (BCG-) administered mouse [1]. Additionally, we previously reported that subcutaneous administration of Z-100 to mice increased the number of CD4^+^T, CD8^+^T, natural kill (NK), and NKT cells and the production of granzyme B in the inguinal lymph nodes [2]. In another study, we also showed that Z-100 induced tumor necrosis factor- (TNF-) *α* production from the mouse macrophage cell line Raw264.7. However, some aspects of Z-100 have not yet been fully investigated, including the detailed mechanisms of each elicited immune response and the key components involved.

Macrophages are ubiquitous in the body and are highly plastic cells with many functions, including tissue development and homeostasis, removal of cellular debris, elimination of pathogens, and regulation of inflammatory responses [3]. While the subtype classification of macrophages is controversial, they are generally divided into M1 (classically activated macrophages) and M2 (alternatively activated macrophages) types. Polarization to M1 macrophages occurs in the presence of granulocyte-macrophage colony-stimulating factor (GM-CSF), IFN-*γ*, TNF-*α*, lipopolysaccharide (LPS), or other pathogen-associated molecular patterns and is thought to contribute to antitumor immunity [4]. Meanwhile, polarization to M2 macrophages occurs in the presence of macrophage colony-stimulating factor (M-CSF), IL-4, IL-10, IL-13, transforming growth factor- (TGF-) *β*, glucocorticoids, or immune complexes and is thought to promote tumor progression [4].

In this study, we used mouse bone marrow cells and human CD14^+^ cells, which are known to differentiate into macrophage-like cells in the presence of GM-CSF [5–7], to study the mechanisms by which Z-100 induces cytokine production. We also sought to identify the active components of Z-100. Previous reports indicate that Z-100 is mainly composed of neutral sugars [8, 9]. Immunostimulants derived from bacteria containing the neutral sugars *α*-mannan, *β*-glucan, lipomannan, and lipoarabinomannan have been reported [10, 11]. C-type lectin receptors including Dectin-1, Dectin-2, mannose receptor, Mincle, SIGNR3, and DC-SIGN are known receptors for these sugars [11]. In our previous report, we suggested that peptidoglycan- (PG-) like components of Z-100 may be involved in TNF-*α* production [8]. In the present study, we found that Z-100 harbors PG-like components, through which it increases levels of IL-12p40. In addition, we studied the effect of Z-100 on cytokine production *in vivo* to clarify the initiation of its immunomodulatory effects.

## 2. Materials and Methods

### 2.1. Mice

All mouse experimental protocols were approved by the Animal Ethics Committee of Zeria Pharmaceutical Co., Ltd. Male C57BL/6 mice purchased from the Jackson Laboratory Japan (Yokohama, Japan) were housed in pathogen-free conditions with food and water available ad libitum.

### 2.2. Reagents

Z-100 was produced by Zeria Pharmaceutical Co., Ltd. (Tokyo, Japan). Pam3CSK4 was purchased from Novus Biologicals (CO, U.S.A.). Muramyl dipeptide (MDP) and PG were purchased from InvivoGen (CA, U.S.A.). Spleen tyrosine kinase inhibitor IV (Syk inhibitor) was purchased from Merck (Darmstadt, Germany).

### 2.3. Treatment of Bone Marrow-Derived Macrophages (BMDM)

Eight-week-old C57BL/6 mice were sacrificed by cervical dislocation, and bone marrow cells were collected. The bone marrow cells were cultured in RPMI1640 medium (Thermo Fisher Scientific, MA, U.S.A.) containing 10% fetal bovine serum (FBS, Hyclone Laboratories, UT, U.S.A.), 200 U/mL penicillin (Meiji Seika Pharma, Tokyo, Japan), 200 *μ*g/mL streptomycin (Meiji Seika Pharma, Tokyo, Japan), and 20 ng/mL murine GM-CSF (PeproTech, NJ, U.S.A.) at 37°C in 5% CO_2_. On the second day of culture, 75% of the culture medium was replaced, and on the fourth day, all of the medium was replaced. On the seventh day, cells adhering to the dish were collected with a scraper. The cell suspension (2 × 10^5^/100 *μ*L/well) was added to collagen-coated 96-well plates (Becton Dickinson and Company, NJ, U.S.A.) and incubated for 30 minutes with 20 ng/mL murine GM-CSF. After 30 minutes, the culture supernatant was removed, and each stimulant was added to the above medium containing 0.3 ng/mL LPS without GM-CSF. Medium without LPS was used for Pam3CSK4 stimulation. The culture supernatant was collected for further experiments.

### 2.4. Treatment of Human CD14^+^ Cells

Human CD14^+^ cells were purchased from Lonza (MD, U.S.A.). Lot No. 0000347745 was used in the nucleotide-binding oligomerization domain 2 (NOD2) siRNA experiment, and Lot No. 1F4563 was used in the other experiments. Frozen human CD14^+^ cells in a cryovial were thawed and cultured in RPMI1640 medium containing 10% FBS, 200 U/mL penicillin, 200 *μ*g/mL streptomycin, 2 mM L-glutamine (Thermo Fisher Scientific, MA, U.S.A.), and 50 ng/mL human GM-CSF (PeproTech, NJ, U.S.A.) at 37°C in 5% CO_2_ (1 × 10^5^/150 *μ*L/well, 96-well plate). On day 5 and day 6, the old medium was replaced with a new one. On day 7, each stimulant was added to the medium described above modified to contain 5% FBS without GM-CSF. One day later, the culture supernatant was collected.

In the NOD2 siRNA experiment, the medium was changed on day 5, and on day 6, the cells were collected using trypsin-EDTA (Sigma-Aldrich, MO, U.S.A.). The cells were resuspended in the medium described above modified to contain 5% FBS without GM-CSF and seeded onto siRNA custom plates (6 × 10^4^/100 *μ*L/well) produced by CytoPathfinder (Tokyo, Japan). The siRNAs (NOD2 siRNA#1: CTGCCACATGCAAGAAGTATA, NO2 siRNA#2: TTGCGCGATAACAATATCTCA) used to make the siRNA custom plates were purchased from QIAGEN (Hilden, Germany). Two days later, each stimulant was added, and one day later, the culture supernatant was collected.

### 2.5. Quantification of Cytokines by ELISA

All mouse and human cytokines were measured using ELISA kits purchased from R&D Systems (MN, U.S.A.), according to the manufacturer's instructions.

### 2.6. Preparation of Anion and Nonanion Fractions

Z-100 was added to a centrifugal ultrafiltration unit (Amicon ultra 3 kDa, Merck Millipore, MA, U.S.A.) and centrifuged. The retentate was collected, and 20 mmol/L Tris-HCl (pH 8.0) was added. The resulting liquid was passed through a HiTrap QFF column (GE Healthcare, IL, U.S.A.), and the flowthrough was defined as the nonanion fraction. Next, an elution buffer (1 mol/L NaCl, 20 mmol/L Tris-HCl, pH 9.0) was added to the column, and the eluted liquid was defined as the anion fraction. Using Amicon ultra 3 kDa, the elution buffer in the nonanion and anion fractions was replaced with distilled water.

### 2.7. Analysis of Amino Acids, Amino Sugars, Neutral Sugars, and PGs

For amino acid analysis, samples were acid-hydrolyzed with 6 M hydrochloric acid (HCl) for 22 hours at 110°C. For analysis of amino sugars (containing muramic acid), samples were acid-hydrolyzed with 4 M HCl for 6 hours at 100°C. For analysis of neutral sugars, samples were acid-hydrolyzed with 2 M trifluoroacetic acid for 6 hours at 100°C. Subsequently, each amino acid (containing muramic acid) was analyzed using an Amino Acid Analyzer L-8900 (Hitachi, Tokyo, Japan). Amino sugars (without muramic acid) and neutral sugars were measured by high-performance liquid chromatography (HPLC). To analyze PG fragments, first Z-100 and the anion fraction were added to the 3 kDa centrifugal filter unit and centrifuged. The retentate was collected and enzymatically digested with 5000 U mutanolysin (Sigma-Aldrich, MO, U.S.A.) for 16 hours at 37°C. Thereafter, the samples were heated at 100°C for 5 minutes, added to a 10 kDa centrifugal filter unit (Merck Millipore), and centrifuged to collect the filtrate. These samples were measured by LC-MS/MS. The anion fraction was measured at 100-fold concentration, and the Z-100 was measured at 20-fold concentration.

### 2.8. Cytokine Expression *In Vivo*

Z-100 was administered subcutaneously at 1 mg/kg into the right inguinal region of 8-week-old C57BL/6 mice once a day for 1 week. One week later, the mice were sacrificed by cervical dislocation and the skin at the administration site (right side) and the skin at the same location on the opposite side of the body (left side) were collected. Total RNA was prepared from the skin samples using a ReliaPrep™ RNA Tissue Miniprep System (Promega, WI, U.S.A.) in accordance with the manufacturer's instructions. The quantity and quality of the purified total RNA samples were checked by measuring the optical density of each sample at 260 nm and 280 nm. The total RNA was subsequently converted to cDNA using the High Capacity RNA-to-cDNA Kit (Life Technologies, CA, U.S.A.). Predesigned glyceraldehyde-3-phosphate dehydrogenase (GAPDH, ID: Mm99999915_g1), IL-12p40 (ID: Mm01288992_m1), TNF-*α* (ID: Mm00443258_m1), or IL-1*β* (ID: Mm00434228_m1) TaqMan probes/primer sets (Life Technologies) and Absolute qPCR ROX Mix (Thermo Fisher Scientific) were added to the cDNA, and polymerase chain reaction (PCR) was performed using the ABI PRISM 7900HT Sequence Detection System (Applied Biosystems, MA, U.S.A.). The data were normalized to GAPDH gene expression and subjected to calculations using the comparative *C*_*T*_ method according to ABI User Bulletin.

### 2.9. Statistical Analysis

*P* values less than 0.05 were considered statistically significant. In multiple comparisons, the homoscedasticity of the groups was first evaluated using Bartlett's test. When the groups were homoscedastic, the *P* value was calculated using parametric Dunnett's multiple comparisons test. When the groups were not homoscedastic, the *P* value was calculated using nonparametric Steel's multiple comparisons test. In the two-group comparison, the homoscedasticity of the groups was first evaluated using an *F*-test. When the groups were homoscedastic, the *P* value was calculated using Student's *t*-test. When the groups were not homoscedastic, the *P* value was calculated using Aspin-Welch's *t*-test. In the time course comparison, the *P* value was calculated using repeated measures ANOVA.

## 3. Results

### 3.1. Effect of Z-100 on IL-12p40 and IL-1*β* Production from Bone Marrow-Derived Macrophages

**Figure 1:**
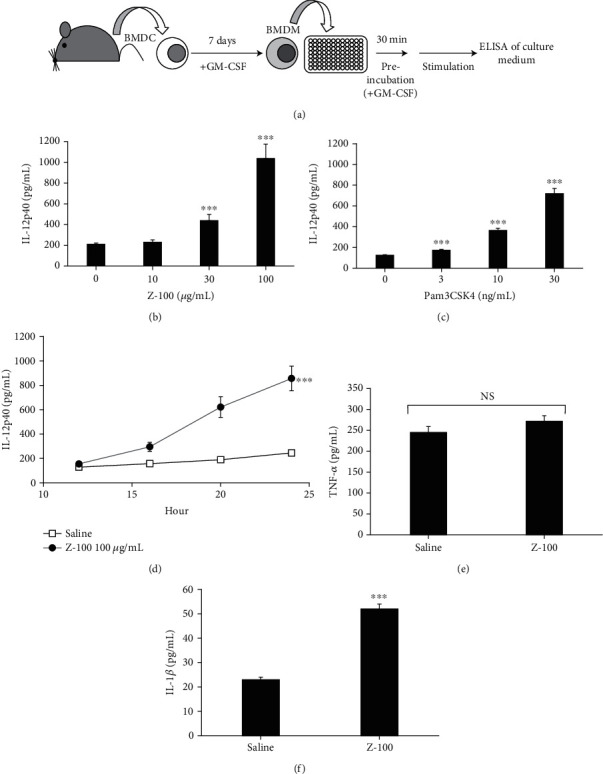
Cytokine production from BMDM following Z-100 stimulation. (a) Schematic of the experimental design. Bone marrow-derived cells were cultured with GM-CSF for 7 days before being seeded onto plates and preincubated for 30 minutes. After preincubation, stimulation was started. (b, c) IL-12p40 concentration in culture supernatant of BMDM after stimulation for 24 hours with Z-100 (b; 10, 30, or 100 *μ*g/mL) or Pam3CSK4 (c; 3, 10, or 30 ng/mL). Symbols indicate a significant difference compared with the 0 *μ*g/mL (saline) group; ^∗∗∗^*P* < 0.001 (Steel's multiple comparisons test). (d) Time course of IL-12p40 production induced by Z-100 (100 *μ*g/mL). Symbols indicate a significant difference compared with the saline group; ^∗∗∗^*P* < 0.001 (repeated measures ANOVA). (e, f) TNF-*α* (e) and IL-1*β* (f) concentration in the culture supernatant of BMDM after stimulation for 24 hours with Z-100 (100 *μ*g/mL). Symbols indicate a significant difference compared with the saline group; ^∗∗∗^*P* < 0.001 (Student's *t*-test). Data show the mean ± S.E.; *n* = 12 per group, pooled from 3 experiments.

A previous study showed that Z-100 enhanced IL-12 production from peritoneal macrophages extracted from mice administered BCG [1]. Further, we previously reported that Z-100 enhanced TNF-*α* production from Raw264.7 cells [8]. In this study, we investigated the effect of Z-100 on macrophages in more detail. Since mouse bone marrow cells are known to differentiate into macrophage-like cells in the presence of GM-CSF [5], we used BMDM differentiation as shown in Figure 1(a). We stimulated BMDM with Z-100 10, 30, or 100 *μ*g/mL and found that Z-100 increased IL-12p40 production in a dose-dependent manner (Figure 1(b)). In addition, Pam3CSK4, a known stimulant of macrophages [12, 13], also increased IL-12p40 in our system (Figure 1(c)). We also examined the time course of IL-12p40 production induced by Z-100 and found that IL-12p40 production increased after 16 hours (Figure 1(d)). The production of other common cytokines produced by macrophages, TNF-*α* and IL-1*β* [4], was also measured. While Z-100 did not significantly increase TNF-*α* production (Figure 1(e)), it did increase IL-1*β*, although production levels were very low (Figure 1(f)).

### 3.2. Effect of Z-100 on IL-12p40, TNF-*α*, and IL-1*β* Production from Human CD14^+^ Cells

**Figure 2:**
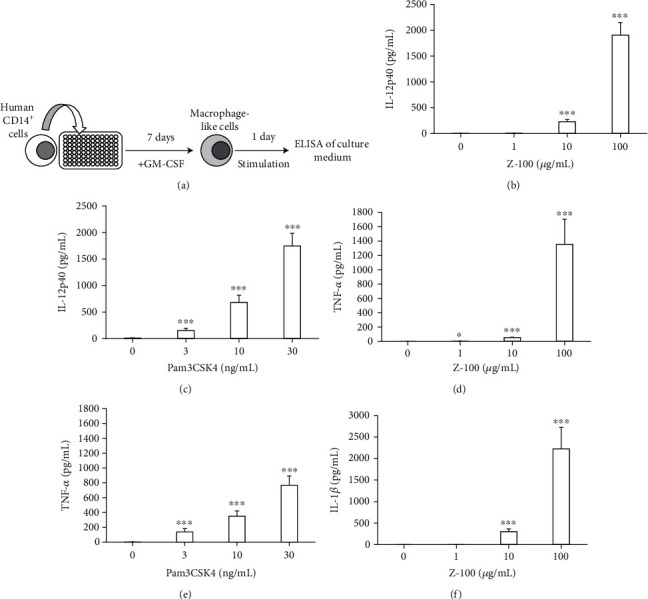
Cytokine production from human CD14^+^ cells after stimulation with Z-100. (a) Schematic of the experimental design. Human CD14^+^ cells were cultured with GM-CSF for 7 days before being stimulated for 24 hours. (b–f) Cytokine concentration in the culture supernatant of human CD14^+^ cells after stimulation with Z-100 (b, d, f; 1, 10, or 100 *μ*g/mL) or Pam3CSK4 (c, e; 3, 10, or 30 ng/mL). Symbols indicate a significant difference compared with the 0 *μ*g/mL (saline) group; ^∗^*P* < 0.05, ^∗∗∗^*P* < 0.001 (Steel's multiple comparisons test). Data show the mean ± S.E.; *n* = 12 per group, pooled from 3 experiments.

It is important to study the effects of drugs such as Z-100 in humans to check their clinical efficacy. As the immune response of human cells to Z-100 requires further investigation, we examined Z-100-mediated cytokine production from human CD14^+^ cells. Human CD14^+^ cells are known to differentiate into macrophage-like cells in the presence of GM-CSF [6, 7]. As shown in Figure 2(a), human CD14^+^ cells were cultured with GM-CSF for 7 days before being stimulated for 24 hours. Z-100 was then added, and after 24 hours, the IL-12p40 concentration in the culture supernatant was measured by ELISA. Stimulating CD14^+^ cells with 10 *μ*g/mL or higher doses of Z-100 increased production of IL-12p40 (Figure 2(b)). Pam3CSK4, which was also used as a general stimulant, likewise increased production of IL-12p40 (Figure 2(c)). Unlike our results in mouse BMDM, Z-100 increased TNF-*α* production in human CD14^+^ cells (Figure 2(d)). Further, Z-100 also significantly increased IL-1*β* production, and levels were higher than those observed in mouse BMDM (Figure 2(f)). While Pam3CSK4 also increased production of TNF-*α* (Figure 2(e)), it did not affect production of IL-1*β* (data not shown).

We also compared CD14^+^ cells between two different donors. We found that while IL-12p40 levels showed a similar trend, reactivity differed between donors (Supplementary Figure 1(a)). We also observed large differences in TNF-*α* and IL-1*β*, with cytokine production by CD14^+^ cells from one donor being much lower than that of the other (Supplementary Figures 1(b) and 1(c)). Additionally, we observed comparable trends in IL-12p40 in three other donors (data not shown); however, as these findings were obtained on a different day from those described above, they may not be directly comparable.

### 3.3. Identification of PG Fragments in Z-100

**Figure 3:**
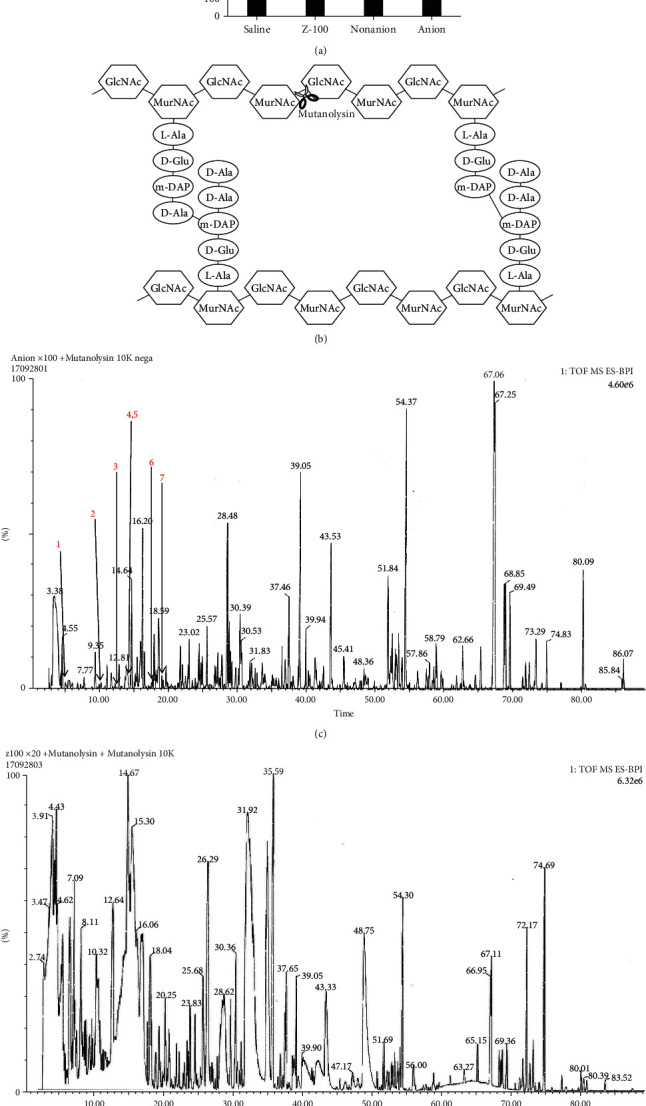
PG fragments in Z-100 and their effect on IL-12p40 concentration. (a) IL-12p40 concentration in the culture supernatant of BMDM after stimulation for 24 hours with Z-100 (100 *μ*g/mL), nonanion fraction, or anion fraction. Symbols indicate a significant difference compared with the saline group; ^∗∗∗^*P* < 0.001 (Steel's multiple comparisons test). Data show the mean ± S.E.; *n* = 16 per group, pooled from 4 experiments. (b) General PG structure of *Mycobacterium tuberculosis* and digestion site of mutanolysin. Mutanolysin cleaves the bond between MurNAc and GlcNAc. (c, d) LC-MS/MS analysis of the anion fraction (c) and Z-100 (d). The anion fraction was measured at 100-fold concentration, and Z-100 was measured at 20-fold concentration.

Based on the reactivity in mice and differences between donors in humans, we concluded that IL-12p40 had the most stable response to Z-100 of the three cytokines examined. Therefore, we next sought to identify the components in Z-100 that were responsible for its effects on IL-12p40. Since the active components of Z-100 were presumed to be neutral sugars or PG-like components, we used an ion-exchange column (HiTrap QFF column) to separate them into anionic and nonanionic fractions. By stimulating BMDM with these fractions, we found that the anionic fraction increased IL-12p40 production, while the nonanionic fraction did not (Figure 3(a)). To identify the composition of these fractions, amino acids, amino sugars, and neutral sugars were measured (Table 1). As shown in Table 1, the main components of Z-100 were neutral sugars, while amino acids made up only a small proportion. Among the two fractions, the nonanion fraction contained a large amount of neutral sugars, while the anion fraction contained a relatively large amount of amino acids. Interestingly, the anion fraction contained a relatively large amount of glutamic acid, alanine, diaminopimelic acid, glucosamine, and muramic acid, which are known to be constituents of PG (Figure 3(b)). Therefore, to investigate whether it contains PG fragments, the anion fraction was enzymatically digested with commercially available muramidase (mutanolysin). Mutanolysin cleaves the bond between N-acetylmuramic acid (MurNAc) and N-acetylglucosamine (GlcNAc) to produce monomeric PG fragments (Figure 3(b)). We conducted LC-MS/MS analysis of the digested anion fraction and found that the mass spectrum (Figure 3(c)) showed some peaks that correlated with PG fragments (Table 2). In contrast, after enzymatically digesting Z-100 with mutanolysin and performing LC-MS/MS analysis, no clearly separated peaks were observed (Figure 3(d)).

### 3.4. Effect of PG and MDP on IL-12p40

**Figure 4:**
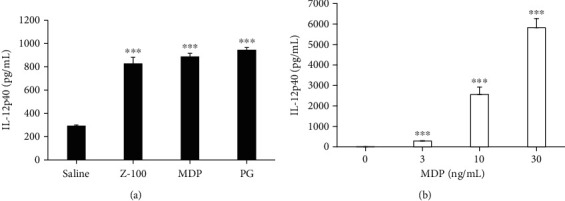
IL-12p40 production after stimulation with MDP and PG. (a) IL-12p40 concentration in the culture supernatant of BMDM after stimulation for 24 hours with Z-100 (100 *μ*g/mL), MDP (100 ng/mL), or PG (2 *μ*g/mL). Data show the mean ± S.E.; *n* = 16 per group, pooled from 4 experiments. (b) IL-12p40 concentration in the culture supernatant of human CD14^+^ cells after stimulation for 24 hours with MDP (3, 10, or 30 ng/mL). Data show the mean ± S.E.; *n* = 10-12 per group, pooled from 3 experiments. Symbols indicate a significant difference compared with the saline or 0 ng/mL group; ^∗∗∗^*P* < 0.001 (Steel's multiple comparisons test).

After finding that the anion fraction increased IL-12p40 production, we predicted that the fraction contained PG. Therefore, we investigated whether commercially available PG also increases IL-12p40 production from macrophages. We also examined the effect of MDP, the minimal bioactive structure of PG. As shown in Figure 4(a), PG and MDP increased IL-12p40 production from BMDM in a dose-dependent manner, similar to Z-100 (Supplementary Figures 2(a) and 2(b)). Stimulation of human CD14^+^ cells with MDP also resulted in a dose-dependent increase in IL-12p40 production (Figure 4(b)).

### 3.5. NOD2-Mediated Effect of Z-100 on IL-12p40

**Figure 5:**
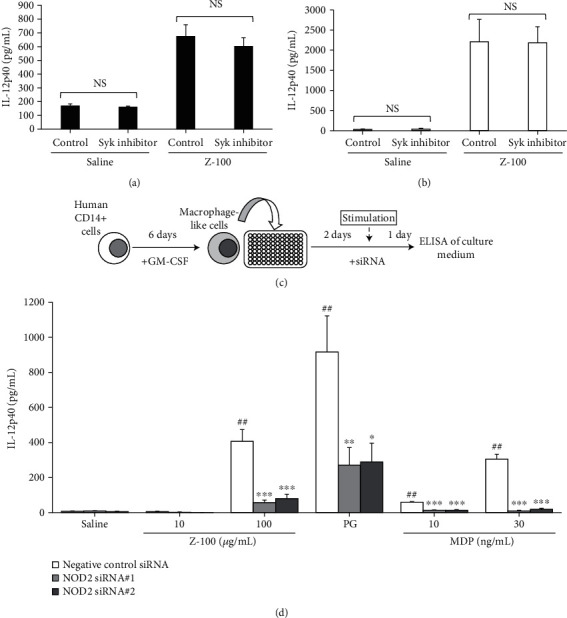
NOD2-mediated effect of Z-100 on IL-12p40. (a) IL-12p40 concentration in the BMDM culture supernatant in the presence of Z-100 (100 *μ*g/mL) and a Syk inhibitor (100 nM). Data show the mean ± S.E.; *n* = 12 per group, pooled from 3 experiments. Statistical analysis was performed using Student's *t*-test (vs. control; NS: not significant). (b) IL-12p40 concentration in the human CD14^+^ cell culture supernatant in the presence of Z-100 (100 *μ*g/mL) and a Syk inhibitor (100 nM). Data show the mean ± S.E.; *n* = 7 per group, pooled from 2 experiments. Statistical analysis was performed using Student's *t*-test (vs. control; NS: not significant). (c) Schematic of the knockdown experimental design. Human CD14^+^ cells were cultured with GM-CSF for 6 days before being seeded onto siRNA custom plates (NOD2 siRNA#1, NOD2siRNA#2, and negative control siRNA) and incubated for 48 hours. After 48 hours, stimulants were added to the medium, and the cells were cultured for 24 hours. (d) IL-12p40 concentration in the culture supernatant of human CD14^+^ cells with siRNAs. The cells were stimulated with Z-100 (10 or 100 *μ*g/mL), PG (2 *μ*g/mL), or MDP (10 or 30 ng/mL). Data show the mean ± S.E.; *n* = 9 per group, pooled from 3 experiments. ^##^*P* < 0.01 vs. saline treatment in the negative control siRNA transfection group (Steel's multiple comparisons test). ^∗^*P* < 0.05, ^∗∗^*P* < 0.01, and ^∗∗∗^*P* < 0.001 vs. negative control siRNA transfection in each stimulation condition (PG and MDP 10 ng/mL group; Dunnett's multiple comparisons test, other groups; Steel's multiple comparisons test).

As shown in Table 1, Z-100 is mainly composed of neutral sugar components such as arabinose, mannose, and glucose. Therefore, it is possible that *α*-mannan, *β*-glucan, and lipoarabinomannan form components of Z-100 and that they act on C-type lectin receptors [10, 11]. Alternatively, as described above, it is possible that PG is a component of Z-100, in which case its effects may be mediated by NOD2 [10]. First, to investigate the involvement of C-type lectin receptors, we inhibited spleen tyrosine kinase (Syk), which contributes to signaling by these receptors [11]. Syk-mediated inhibition did not suppress the increase in IL-12p40 production induced by Z-100 from either mouse BMDM or human CD14^+^ cells (Figures 5(a) and 5(b)). Next, to inhibit NOD2, we generated two NOD2 siRNAs (#1 and #2) and transfected them into human CD14^+^ cells (Figure 5(c)). As shown in Figure 5(d), both #1 and #2 NOD2 siRNAs suppressed the increase in IL-12p40 induced by Z-100. The NOD2 siRNAs also inhibited the increase in IL-12p40 induced by PG and MDP.

### 3.6. Effects of Z-100 on Cytokines in the Skin of Mice *In Vivo*

**Figure 6:**
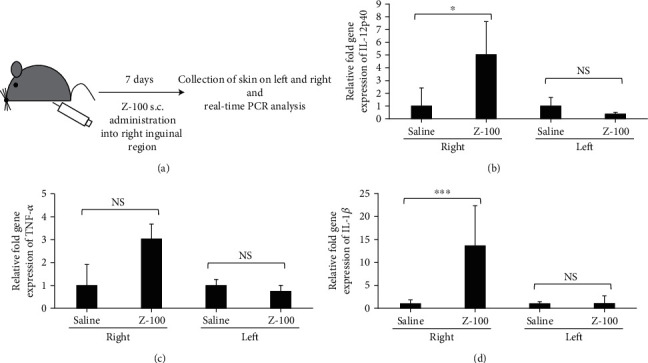
Z-100-mediated increase in cytokine expression *in vivo*. (a) Schematic of the experimental design. After administering Z-100 into the right inguinal region for 7 days, skin samples were collected from the left inguinal region and right inguinal region. (b–d) Relative fold gene expression of IL-12p40 (b), TNF-*α* (c), or IL-1*β* (d) in skin samples from the left and right. Data show the mean ± range; *n* = 4-6 per group. The ranges were determined by evaluating the expression: 2^-*ΔΔ*CT^ with ΔΔCT + S.E. and *ΔΔ*CT–S.E., where S.E. is the standard error of the mean of the *ΔΔ*CT value. TNF-*α* in skin samples from the right and IL-1*β* in skin samples from the left were analyzed using Aspin-Welch's *t*-test; all other analyses were performed using Student's *t*-test (^∗^*P* < 0.05, ^∗∗∗^*P* < 0.001, and NS: not significant vs. saline).

While we successfully investigated the mode of action of Z-100 using cells, as Z-100 is administered subcutaneously in clinical settings, it is important to also investigate the subcutaneous response. Therefore, Z-100 was subcutaneously administered into the right inguinal region of mice for 1 week, and gene expression was measured in skin samples from the left inguinal region and right inguinal region using real-time PCR analysis (Figure 6(a)). Gene expression of IL-12p40 and IL-1*β* significantly increased in the skin surrounding the injection site (Figures 6(b) and 6(d)). Further, TNF-*α* gene expression also showed an increasing trend, although the change was not significant (Figure 6(c)). In contrast, no significant increase was observed in any of the cytokines in the skin sample to which Z-100 was not administered.

## 4. Discussion

This study showed that PG-like components in Z-100 play a role in their ability to increase IL-12p40 production. IL-12 is composed of the heterodimers IL-12p40 and IL-12p35. IL-12 produced by antigen-presenting cells such as macrophages activates the immune system by inducing IFN-*γ* production from T cells and NK cells. IL-12p40 also forms a heterodimer with IL-12p19 and has been suggested to bind to other partners [14].

Oka et al. previously reported that the metastasis-suppressing effect of Z-100 disappears in IL-12p40 KO mice [1]. The authors also reported that Z-100 enhances IL-12 production from peritoneal macrophages extracted from mice administered BCG. However, these macrophages were collected after BCG administration to mice, and as Z-100 is a hot-water extract of *Mycobacterium tuberculosis*, it likely contains some of the same components as BCG and may thus have acted to boost the actions of BCG. Since an experimental system without BCG was required to analyze the effects of Z-100 in detail, we measured the production of IL-12p40 from BMDM without BCG in this study. A recent study reported that BMDM is more immunologically responsive than peritoneal macrophages [15]. Therefore, we expected that BMDM would increase IL-12p40 production in the presence of Z-100 even without BCG. Furthermore, this study also showed that human CD14^+^ cells increase IL-12p40 production in the presence of Z-100. These simple systems for evaluating Z-100 enabled us to conduct a detailed mechanistic analysis. Our findings also suggest that the increase in IL-12p40 induced by Z-100 may be a NOD2-mediated response that arises through Z-100's PG-like components. These results also confirm the involvement of Z-100 in IL-12p40 production, which was not clear from previous studies.

Several methods for analyzing PG have been reported [16–18]. In this study, we used enzymatic digestion with mutanolysin followed by LC-MS/MS analysis to detect PG fragments. The structures shown in Table 2 are similar to those of PG fragments derived from *Mycobacterium tuberculosis* in a previous report [18]. Further, the muramic acids in Table 2 are N-acylated with glycolic acid (MurNGlyc), a known characteristic structure of *Mycobacterium tuberculosis* [19]. Therefore, the PG fragments detected in this study are likely derived from *Mycobacterium tuberculosis*.

In addition to NOD2, PG is also reportedly a ligand for Toll-like receptor (TLR) 2, TLR4, and NOD1 [20, 21]. However, a previous study suggested that the action of PG on TLR may be caused by contamination with lipoproteins and lipoteichoic acids (LTAs) [22]. In the present study, commercial PG had the same enhancement effect on IL-12p40 production as Z-100, as shown in Figure 4, suggesting that Z-100 may act on the same receptor as PG. Further, MDP-mediated IL-12p40 production was similar to that induced by Z-100. Given that MDP is a known ligand for NOD2 but not TLR2, TLR4, and NOD1 [23, 24], we focused on NOD2 in this study and found that knockdown of NOD2 significantly inhibited the increase in IL-12p40 induced by Z-100. Based on this result, Z-100 may have greater specificity for NOD2 than other receptors. We previously reported that while stimulation of Raw264.7 cells with Z-100 or MDP increased TNF-*α* production, stimulation with iE-DAP, a ligand for NOD1, did not [8]. This result likewise suggests that Z-100 does not act on NOD1.

We showed that the main components of Z-100 are neutral sugars such as arabinose, mannose, and glucose. Therefore, it is interesting that the increase in IL-12p40 induced by Z-100 was due to PG-like components and was abolished by NOD2 siRNA. It is clear that neutral sugar-derived immunostimulants are not involved in the Z-100-mediated increase in IL-12p40. In a previous report, we had speculated that the production of TNF-*α* was due to PG-like components [8]; the present analytical results on PGs support that theory. Thus, our present and previous studies provide strong evidence that the PG-like components in Z-100 have immunostimulatory effects. However, polysaccharides in Z-100 may also have such effects, although we have yet to show this. Future studies should examine the actions of polysaccharides in Z-100.

Z-100 is clinically marketed as a subcutaneously administered medicine for leukopenia and is under development as a subcutaneously administered medicine for cancer. However, although its effect on facial skin when administered subcutaneously into the abdominal region has been reported in an atopic dermatitis model [25], there are no reports of the reaction in the skin at the administration site. We showed that IL-12p40 gene expression increased in skin samples into which Z-100 was administered, indicating that Z-100 also increases IL-12p40 *in vivo*. Macrophages are known to exist as antigen-presenting cells in the dermis, where they promote an immune response [26]. Therefore, subcutaneously administered Z-100 may act on macrophages in the skin to increase gene expression of IL-12p40.

Stimulation with Z-100 increased TNF-*α* production from human CD14^+^ cells but not mouse BMDM. However, we previously reported that Z-100 increased TNF-*α* production from Raw264.7 cells [8]. In the same report, we showed that Z-100-mediated TNF-*α* production increased weakly without IFN-*γ*. This finding may be related to the large difference we observed between human CD14^+^ cells from different donors in the present study. We predict that differences between donors may be related to the background of the donor and the condition of the purchased human CD14^+^ cells. In particular, NOD2 has been reported to be induced by stimulation with IFN-*γ* [27], suggesting that the inflammatory condition of the donor may have an effect. Meanwhile, we reported for the first time that Z-100 increases IL-1*β* production. Based on the responses observed in BMDM and human CD14^+^ cells, like TNF-*α*, certain conditions may also be required to affect levels of IL-1*β*. Since IL-12, TNF-*α*, and IL-1*β* are known to be produced by M1 macrophages [4], Z-100 may be involved in the function of M1 macrophages. NOD2 expression is also reportedly increased during cell differentiation into M1 macrophages [28]. Further, there is donor-to-donor variation in the differentiation of CD14^+^ cells into M1 macrophages [29, 30], which may have influenced the results of the present study.

The results of the present study suggest that Z-100 may induce M1 macrophages, which are known to suppress cancer via direct phagocytosis, antigen presentation, and cytokine production [4, 31]. Thus, Z-100 may suppress cancer via M1 macrophage induction. In addition, patients with NOD2 polymorphisms are reportedly at high risk of lymphoma, colorectal cancer, gastric cancer, breast cancer, ovarian cancer, lung cancer, and laryngeal cancer, but at low risk of kidney cancer [32]. Since Z-100 activates NOD2, it is more likely to suppress cancers for which NOD2 polymorphisms are a cancer risk. Thus, the results of the present study may provide insight into which cancer types to target with Z-100 treatment.

In conclusion, we showed that the Z-100-mediated increase in IL-12p40 production arises through its PG-like components via NOD2 receptors. These results will facilitate research into the elucidation of the mode of action of Z-100 and the biological activity of *Mycobacterium tuberculosis*.

## Figures and Tables

**Table 1:** Concentration of amino acids, amino sugars, and neutral sugars in Z-100 and nonanion and anion fractions.

	Concentration (nmol/mL)		
	Z-100	Nonanion	Anion
Aspartic acid	49.42	2.36	10.22
Threonine	51.91	7.68	10.13
Serine	32.98	3.53	6.32
Glutamic acid	88.86	8.06	29.58
Glycine	112.13	6.01	17.27
Alanine	122.91	12.94	39.34
Valine	37.04	3.03	7.76
Methionine	0.71	0.19	0.59
Isoleucine	13.85	0.93	3.02
Leucine	21.02	1.08	4.43
Tyrosine	6.55	0.42	1.95
Phenylalanine	4.19	0.18	0.69
Lysine	14.60	1.44	2.72
Histidine	8.05	0.28	2.23
Arginine	12.40	0.88	3.51
Proline	39.05	5.97	8.02
Diaminopimelic acid	38.33	5.39	18.33

Glucosamine	33.80	7.79	11.49
Galactosamine	18.22	13.44	<2.33
Muramic acid	10.69	1.22	7.09

Arabinose	2951.44	2304.34	101.25
Mannose	6014.23	4843.58	56.06
Glucose	1277.78	725.20	12.77
Galactose	364.02	237.57	37.19

**Table 2:** Predicted structures for peaks 1 to 9 (from Figure 3(c)).

No.	PG fragment	Composition formula	*m*/*z* [M-H] calculated	*m*/*z* [M-H] observed
1	GlcNAc-MurNGlyc-Ala-Glu(NH_2_)-DAP(NH_2_)	C_34_H_58_N_8_O_19_	881.3818	881.3765
2	GlcNAc-MurNGlyc-Ala-Glu(NH_2_)-DAP(NH_2_)-Ala	C_37_H_63_N_9_O_20_	952.4189	952.4339
3	GlcNAc-MurNGlyc-Ala-Glu(NH_2_)-DAP(NH_2_)-Ala-Ala	C_40_H_68_N_10_O_21_	1023.4560	1023.4672
4	GlcNAc-MurNGlyc-Ala-Glu(NH_2_)	C_27_H_45_N_5_O_17_	710.2810	710.2682
5	GlcNAc-MurNGlyc(Anhydro)-Ala-Glu(NH_2_)-DAP(NH_2_)	C_34_H_56_N_8_O_18_	863.3713	863.3717
6	GlcNAc-MurNGlyc(Anhydro)-Ala-Glu(NH_2_)-DAP(NH_2_)-Ala	C_37_H_61_N_9_O_19_	934.4084	934.4144
7	GlcNAc-MurNGlyc(Anhydro)-Ala-Glu(NH_2_)-DAP(NH_2_)-Ala-Ala	C_40_H_66_N_10_O_20_	1005.4455	1005.4597

## Data Availability

Data supporting the findings of this study are available from the corresponding author upon reasonable request.

## Supplementary Materials

Supplementary Materials Supplementary Figure 1: differences between donors of human CD14^+^ cells. Supplementary Figure 2: dose-dependent increase in IL-12p40 from BMDM after stimulation with MDP or PG. Supplementary Table 1: suppression of NOD2 gene expression by NOD2 siRNA in human CD14^+^ cells.
