# Supplementary material for: Peptidoglycan-Like Components in Z-100, Extracted from Mycobacterium tuberculosis Strain Aoyama B, Increase IL-12p40 via NOD2
Source: J Immunol Res. 2022 Jun 22;2022:3530937. doi: 10.1155/2022/3530937 (PMC9242757; doi:10.1155/2022/3530937)
Supplement: Supplementary Materials — Supplementary Figure 1: differences between donors of human CD14+ cells. Supplementary Figure 2: dose-dependent increase in IL-12p40 from BMDM after stimulation with MDP or PG. Supplementary Table 1: suppression of NOD2 gene expression by NOD2 siRNA in human CD14+ cells. [file 3530937.f1.docx]

1. (B)


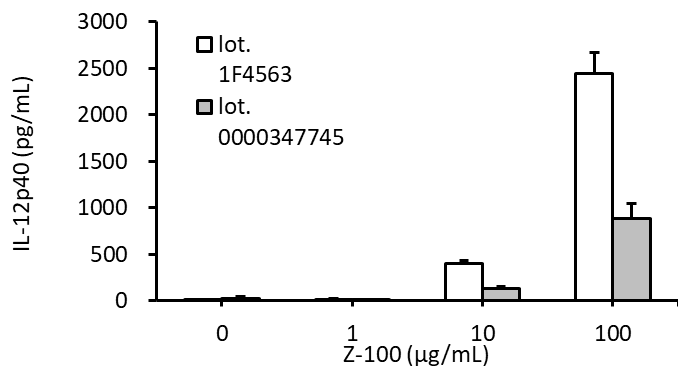

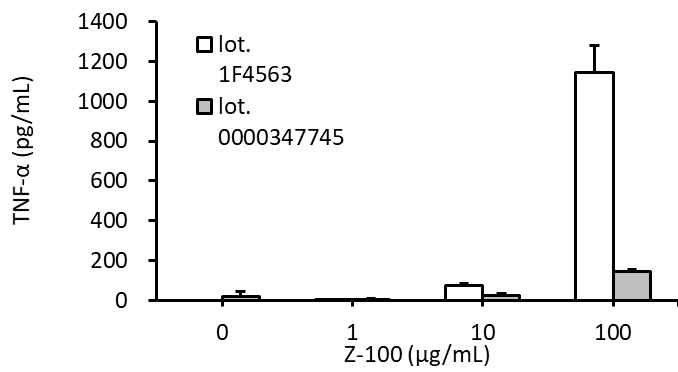


(C)


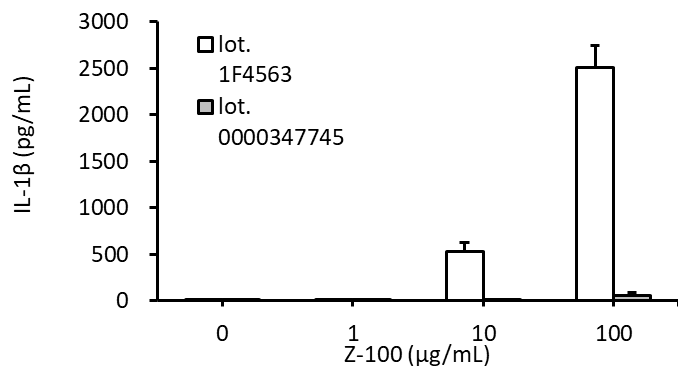


**Supplementary Figure 1: Differences between donors of human CD14^+^ cells.**

(A-C) Cytokine concentration in the culture supernatant of human CD14^+^ cells after stimulation with Z-100 (1, 10 or 100 µg/mL). Human CD14^+^ cells from two donors were cultured with GM-CSF for 7 days before stimulating with Z-100 for 24 hours. Data show mean ± S.E.; n = 3-4.

(A) (B)


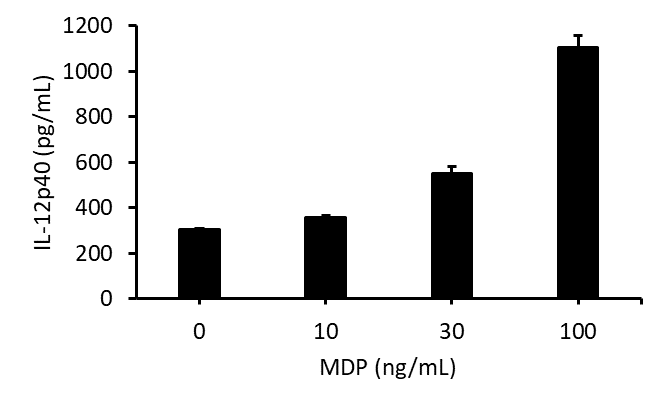

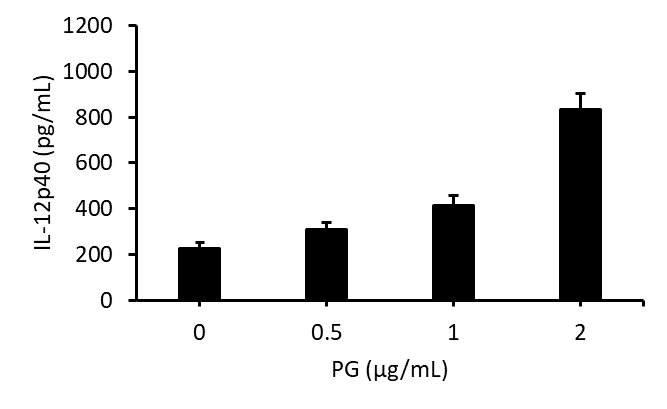


**Supplementary Figure 2: Dose-dependent increase in IL-12p40 from BMDM after stimulation with MDP or PG**

(A-B) IL-12p40 concentration in the culture supernatant of BMDM after stimulating with MDP (A; 10, 30 or 100 ng/mL) or PG (B; 0.5, 1 or 2 µg/mL). Bone marrow-derived cells were cultured with GM-CSF for 7 days before being seeded onto plates and pre-incubated for 30 minutes. After pre-incubation, stimulation was started. Data show mean ± S.E.; n = 4-12.

| Stimulant | Relative fold gene expression of NOD2 | | |
| --- | --- | --- | --- |
|  | Negative control siRNA | NOD2 siRNA#1 | NOD2 siRNA#2 |
| Saline | 1.00 | 0.42 | 0.38 |
| Z-100 10 µg/mL | 1.10 | 0.32 | 0.39 |
| Z-100 100 µg/mL | 0.98 | 0.33 | 0.42 |
| PG | 1.21 | 0.32 | 0.48 |
| MDP 10 ng/mL | 1.08 | 0.25 | 0.32 |
| MDP 100 ng/mL | 0.88 | 0.39 | 0.48 |

**Supplementary Table 1: Suppression of NOD2 gene expression by NOD2 siRNA in human CD14^+^ cells**

Endogenous Control: Human PPIA (Cyclophilin A) Endogenous Control (Life Technologies)

NOD2 TaqMan probes/primer sets: ID Hs00223394_m1 (Life Technologies)
